# Oncogenic Roles of GOLPH3 in the Physiopathology of Cancer

**DOI:** 10.3390/ijms21030933

**Published:** 2020-01-31

**Authors:** Stefano Sechi, Anna Frappaolo, Angela Karimpour-Ghahnavieh, Roberto Piergentili, Maria Grazia Giansanti

**Affiliations:** Istituto di Biologia e Patologia Molecolari del CNR, Dipartimento di Biologia e Biotecnologie, Sapienza Università di Roma, 00185 Roma, Italy; stefano.sechi@cnr.it (S.S.); anna.frappaolo@uniroma1.it (A.F.); angela.karimpourghahnavieh@uniroma1.it (A.K.-G.)

**Keywords:** GOLPH3, Golgi trafficking, tumorigenesis

## Abstract

Golgi phosphoprotein 3 (GOLPH3), a Phosphatidylinositol 4-Phosphate [PI(4)P] effector at the Golgi, is required for Golgi ribbon structure maintenance, vesicle trafficking and Golgi glycosylation. GOLPH3 has been validated as an oncoprotein through combining integrative genomics with clinopathological and functional analyses. It is frequently amplified in several solid tumor types including melanoma, lung cancer, breast cancer, glioma, and colorectal cancer. Overexpression of GOLPH3 correlates with poor prognosis in multiple tumor types including 52% of breast cancers and 41% to 53% of glioblastoma. Roles of GOLPH3 in tumorigenesis may correlate with several cellular activities including: (i) regulating Golgi-to-plasma membrane trafficking and contributing to malignant secretory phenotypes; (ii) controlling the internalization and recycling of key signaling molecules or increasing the glycosylation of cancer relevant glycoproteins; and (iii) influencing the DNA damage response and maintenance of genomic stability. Here we summarize current knowledge on the oncogenic pathways involving GOLPH3 in human cancer, GOLPH3 influence on tumor metabolism and surrounding stroma, and its possible role in tumor metastasis formation.

## 1. The PI(4)P Binding Protein GOLPH3 is An Oncoprotein

Compelling evidence demonstrates that deregulation of intracellular vesicle trafficking contributes to several aspects of cancer phenotypes [1,2]. Golgi phosphoprotein 3 (GOLPH3) has been described as the first example of a Golgi resident oncoprotein [3]. Identified in proteomic-based studies of the Golgi [4,5], GOLPH3 is a highly conserved protein from yeast to humans, which localizes to the trans-Golgi via the direct interaction with PI(4)P, mediated by its unique C-terminal GPP34 domain [6,7]. Localization of GOLPH3 protein to the Golgi is required to maintain Golgi architecture in human cells and *Drosophila melanogaster* [6,8]. 

By using unbiased genome-wide copy number analysis of multiple human solid tumors, Scott and coauthors [9] identified a frequent 5p13 amplification in several human solid tumors including melanoma, colon adenocarcinoma and non-small-cell lung cancer. Of the four genes within the 5p13 genomic region, GOLPH3 was shown as the gene targeted for activation in cancers as its depletion was found to revert oncogenic transformation in cell culture. GOLPH3 cooperates with activated HRAS^V12^ to increase transformed focus formation in *Ink4a/Arf*-deficient primary mouse embryonic fibroblasts [9]. Moreover, GOLPH3 up-regulation was shown to significantly enhance mouse xenograft tumor growth in vivo. Overall these data validated GOLPH3 as a bona fide oncogene [9]. Since this study, over 30 research papers correlated GOLPH3 overexpression to several cancers including melanoma, lung cancer, breast cancer, glioma, and colorectal cancer [10,11]. Several papers provided evidence that GOLPH3 promotes cell proliferation and tumorigenicity of MDA-MB231 and MCF-7 breast cell lines and U251 and U87 glioblastoma cell lines [12,13,14,15]. Frequent overexpression of GOLPH3 and correlation with poor prognosis have been observed in multiple tumor types including 52% of breast cancers [13] and 41 to 53% of glioblastoma [14,15]. These findings indicated a role of GOLPH3 in cancer progression.

The oncogenic activity of GOLPH3 has been primarily correlated with its ability to influence signaling downstream of the mammalian target of rapamycin (mTOR), resulting in enhanced activation of both mammalian TORC1 and TORC2 complexes [9,10]. This idea was supported by preclinical treatment studies showing that GOLPH3-expressing tumors are much more sensitive to mTOR inhibitors, raising the possibility that GOLPH3 levels might predict rapamycin efficacy in tumor therapy. However, the mechanistic basis that links the mTOR pathway to GOLPH3 remains to be elucidated, and multiple molecular mechanisms have been suggested to explain how GOLPH3 drives cancer [16,17].

**Figure 1:**
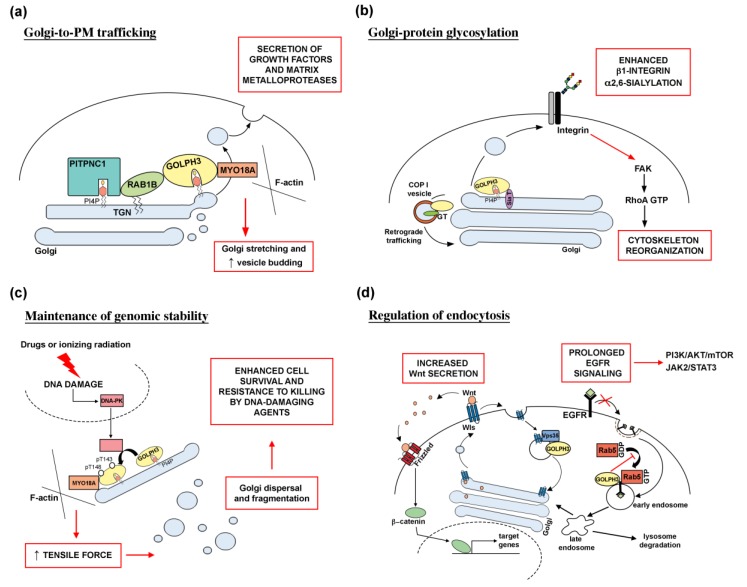
Molecular mechanisms underlying GOLPH3 function in human cancer. (**a**) Altered Golgi-to-PM trafficking. Recruitment of the PI(4)P-binding protein GOLPH3 at the trans-Golgi network (TGN), requires the PITPNC1/RAB1B network. Overactivation of the GOLPH3/MYO18A pathway leads to Golgi extension and enhanced vesicle release, resulting in the massive secretion of growth factors and matrix metalloproteases, which are responsible for cancer malignancy. (**b**) GOLPH3 role in Golgi-protein glycosylation. GOLPH3 is involved in COPI-mediated retrograde trafficking of several Golgi glycosyltransferases (GT). GOLPH3 protein binds to sialyltransferases (Sia T); its overexpression results in enhanced α2,6-sialylation of β1-integrins. Altered glycosylation affects integrin-mediated signaling pathway, leading to activation of FAK (focal adhesion kinase) and RhoA-dependent actin cytoskeleton reorganization and cell migration. (**c**) GOLPH3 is required for Golgi ribbon reorganization in response to DNA damage. DNA damage, induced by treatment with drugs and ionizing radiation, leads to activation of the DNA damage protein kinase (DNA-PK) that phosphorylates GOLPH3 on T143 and T148 residues. In turn, phosphorylation of GOLPH3 increases its interaction with MYO18A and its tensile force on the Golgi, leading to fragmentation of Golgi membranes and their dispersal throughout the cytoplasm. Massive Golgi fragmentation has been correlated to tumor development and maintenance since it is linked to cell survival and resistance to killing by DNA-damaging agents. (**d**) Relationship between GOLPH3 oncogenic activity and endocytic trafficking. GOLPH3 associates with the retromer complex subunit Vps35 to regulate recycling of several transmembrane receptors including the Wnt chaperone Wntless (Wls). In glioma cell lines, GOLPH3 overexpression increases Wls recycling and Wnt secretion, leading to abnormal Wnt/β-catenin signaling. GOLPH3 oncogenic activity in endocytic trafficking is responsible for a prolonged EGFR signaling. GOLPH3 binds to Rab5 and inhibits its activation on early endosome. High levels of GOLPH3 protein impair Rab5-mediated endocytosis and degradation of EGFR. As a result, overactivation of downstream signaling pathways such as PI3K/AKT/mTOR and JAK2/STAT3 promote cell proliferation and tumor transformation.

This review will highlight the cellular processes that require GOLPH3 protein and the molecular pathways involved in GOLPH3-driven oncogenic transformation and metastasis in tumors (Figure 1).

## 2. GOLPH3, a PI(4)P Effector that Plays Essential Roles in Golgi-to-Plasma Membrane Trafficking

PI(4)P is highly enriched in the cytosolic face of the trans-Golgi membranes [18,19], where the levels of this lipid are maintained by the concerted actions of Golgi PI(4)-kinases and Sac1 Phoshoinositide phosphatase [20]. Experimental studies in different cell systems have demonstrated the requirement for PI(4)P for Golgi structure and secretion via the Golgi [21,22,23,24]. Localization of GOLPH3 to Golgi membranes requires its binding to PI(4)P in several organisms such as humans, yeast (for the ortholog Vps74p) and *Drosophila* [6,8,25]. The function of GOLPH3 is required to maintain Golgi ultrastructure and Golgi-to-plasma membrane (PM) trafficking [6,8]. Dippold and coauthors [6] demonstrated that Golgi architecture in human cells depends on the tight interaction of GOLPH3 with unconventional Myosin 18A (MYO18 A) and PI(4)P, which connect the Golgi to F-actin cytoskeleton. Several papers showed that the PI(4)P-GOLPH3/MYO18A/F-actin module mediates a tensile force that stretches the Golgi membranes and contributes to the characteristic Golgi shaping and vesicle budding for forward trafficking [6,26,27,28]. Depletion of GOLPH3 or MYO18A impairs Golgi-to-PM trafficking of vesicular stomatitis virus G glycoprotein [6,29,30] and exit of Hepatitis C virus from infected cells [27]. Moreover, overall endogenous secretion, measured by unbiased pulse-chase experiments, is severely affected by the knockdown of GOLPH3 or MYO18A [26]. Recent data showed that GOLPH3 alone (independently from MYO18A) is able to induce PI(4)P-dependent membrane curvature by insertion of a hydrophobic-dependent β-loop into the membrane bilayer [30]. However, the ability of GOLPH3 protein to induce membrane curvature at the Golgi, although essential for efficient Golgi-to-PM trafficking, does not appear sufficient to support forward trafficking. Blocking the interaction of GOLPH3 with its binding partner MYO18A results in extensive curvature of Golgi membranes, dramatic tubulation of the Golgi and ineffective trafficking. Just like GOLPH3, MYO18A is a driver of human cancers [16,28,31], and several have papers indicated that defective Golgi-to-PM trafficking might contribute to GOLPH3-driven malignant secretory phenotypes [29,32,33]. Halberg and coauthors [32] correlated GOLPH3 Golgi function with the malignant secretion of PITPNC1 oncoprotein. PITPNC1 gene is amplified in a large fraction of breast cancers and its overexpression promotes metastatic progression of breast, melanoma and colon cancers [32]. By binding Golgi-resident PI(4)P, PITPNC1 protein localizes Rab1B to the Golgi and contributes to Golgi extension and enhanced vesicular release via the recruitment of GOLPH3 (Figure 1). The PITPNC-Rab1B-GOLPH3 Golgi network drives malignant secretion of growth factors and matrix metalloproteases, which in turn lead to increased cell motility, extracellular matrix remodeling, metastasis, and angiogenesis [32]. 

On the other hand, the PI(4)P/GOLPH3/MYO18A/F-actin pathway is required to drive reorientation of the Golgi in wound healing and directional trafficking toward the front of the cell, with an important implication for cell migration to promote cancer invasion and metastasis [33].

## 3. GOLPH3 Role in Golgi-Protein Glycosylation

The oncogenic secretion properties of GOLPH3 are not limited to its role in anterograde Golgi-to-PM trafficking. Another route through which GOLPH3 is thought to influence cell transformation, is the retrograde intra-Golgi trafficking of protein glycosyltransferases (Figure 1) [10,25,34]. 

Experimental data from both yeast and human cells indicate that GOLPH3 facilitates COPI-mediated intra-Golgi trafficking of several Golgi glycosyltransferases including human α2,6-sialyltransferase I [34,35,36,37]. Isajii and coauthors reported that human GOLPH3 binds to sialyltransferases and affects sialylation of N-glycans, especially α2,6-sialylation [36]. Notably, previous work showed increased α2,6-sialylation of β1-integrins in several transformed cell types and reported a correlation between N-glycosylation of integrins and the epithelial-mesenchymal transition (EMT) that contributes to cancer metastasis [38,39]. Metastasis, the process in which cancer cells spread from the primary tissue to the surrounding tissues, requires changes in cell-cell adhesion and gain of invasion/cell migration capacity [40]. The EMT represents a critical event in cancer progression in which epithelial cells acquire the mesenchymal phenotype, with motile and invasive characteristics [40]. Isaji et al. [36] demonstrated the importance of GOLPH3-dependent sialylation on N-glycans, including those on β1-integrins, and reported that overexpression of α2,6-sialyltransferase-I (SiaT) is able to rescue integrin-dependent cell migration alterations caused by GOLPH3 depletion (Figure 1). The link between GOLPH3 and protein glycosylation was also reported by Eckert and coauthors [35]. They showed that GOLPH3 protein directly interacts with Core 2N-acetylglucosaminyltransferase (C2GnT) and SiaT, and that the incorporation of C2GnT and SiaT in COPI-coated vesicles is dependent on GOLPH3 function. 

Alterations in protein glycosylation not only represent a hallmark feature of malignant transformation and progression, but also provide novel diagnostic and therapeutic targets [39]. The most frequent changes in protein glycosylation in cancer cells include high mannose N-linked glycans, altered sialylation or fucosylation of N- and O-linked glycans, and expression of cancer-specific glycan structures such as Tn/ STn antigens [39]. 

Altered glycan structures can also influence endocytosis and recycling of transmembrane receptors resulting in prolonged growth factor signaling [3]. Taken together, these data indicate that the role of GOLPH3 in glycosylation is an important aspect of its oncogenic properties.

## 4. GOLPH3 and Maintenance of Genomic Stability

Errors in DNA damage response contribute to genome instability, a defining feature of cancer cells that generates their intratumoral heterogeneity and facilitates tumor progression [41]. A number of studies provided evidence that GOLPH3 is required for maintenance of genomic stability. Farber-Katz and colleagues characterized a role for GOLPH3 for Golgi ribbon reorganization in response to the DNA damage induced by treatment with camptothecin, doxorubicin and ionizing radiation [29]. In response to a perturbation causing DNA damage, DNA damage protein kinase (DNA-PK) phosphorylates GOLPH3 on Thr143 and Thr148 in a TQ motif. In turn, phosphorylation of GOLPH3 increases its interaction with MYO18A and its tensile force on the Golgi, leading to Golgi fragmentation and their dispersal throughout the cytoplasm. Remarkably, the study of Farber-Katz clearly showed that the DNA-PK/GOLPH3/MYO18A pathway is necessary for cell survival of cancer cells after DNA damage, indicating that Golgi fragmentation may contribute to tumor development and maintenance (Figure 1). A more recent study from Ognibene and collaborators [42] analyzed the effects of curcumin on DNA damage in two human neuroblastoma cell lines, reporting an increase of GOLPH3 expression accompanied by Golgi fragmentation and increased expression of TPX2 oncoprotein. These data suggest that a novel cancer therapeutic strategy should combine molecules able to interfere with GOLPH3 and TPX2 pathways with standard DNA damaging therapeutic agents. 

Work from our group involved GOLPH3 protein in cytokinesis, another cellular process that contributes to maintain genomic stability [8]. *Drosophila* GOLPH3 accumulates at the cleavage furrow of dividing spermatocytes and larval neuroblasts, and loss of this protein results in cytokinesis failures. GOLPH3 interacts with components of the cytokinetic machinery and vesicle trafficking proteins. Moreover, GOLPH3 function in cytokinesis is intimately connected to its ability to bind PI(4)P, suggesting its key action in coupling PI(4)P signaling and membrane trafficking with actomyosin ring dynamics. The involvement of GOLPH3 in cytokinesis is highly relevant to cancer biology because cytokinesis failures lead to tetraploidy, which promotes tumorigenesis and chromosome instability (CIN), accelerates cancer heterogeneity and evolution, contributes to increased resistance to drug treatment, and has been correlated with poor prognosis in colon cancer patients [43,44,45,46]. Moreover, recent work suggests that causing polyploidy by cytokinesis failure could provide an effective strategy to selectively block cell division in cancer cells [47]. Thus, a thorough analysis of GOLPH3 function and regulation in cytokinesis may open up new avenues to treat GOLPH3-overexpressing cancers. 

## 5. GOLPH3 and Regulation of Endocytosis

Substantial evidence correlates the oncogenic activity of GOLPH3 with its role in endocytic trafficking (Figure 1). Consistent with a role in endocytic trafficking, GFP-GOLPH3 protein was visualized on endosomes and on the plasma membrane [4]. Moreover, human and *Drosophila* GOLPH3 proteins interact with the Vps35 protein [8,9], a component of the retromer, an endosomal complex that orchestrates several vesicle trafficking routes including trafficking between the endosomes and trans-Golgi network (TGN), and endosome-to-plasma membrane transport [48,49]. Several papers revealed the requirement for a retromer for proper secretion of Wnt morphogen in *Drosophila melanogaster* by controlling the recycling of its sorting receptor [50,51,52]. Moreover, loss of *Drosophila* Vps35 in blood cells was shown to impair endocytosis of several transmembrane proteins leading to enhanced plasma membrane localization of Epidermal Growth Factor receptor (EGFR) and platelet-derived growth factor, and a corresponding increase of downstream signaling [53]. Based on these data, it has been suggested that GOLPH3 might affect the retromer-mediated recycling of the cell surface receptors including the receptor tyrosine kinases, leading to prolonged downstream signaling which influences cancer-relevant activities [3]. A correlation between endocytic trafficking and GOLPH3-driven oncogenesis was suggested by a recent paper from Zhou and coauthors [54], which reported a role for GOLPH3 in glioma progression for inhibiting endocytosis and degradation of EGFR. 

## 6. GOLPH3 and Tumor-Stromal Interaction

Data from Listanti and collaborators identified the requirement for GOLPH3 for controlling cancer metabolism [55,56]. Their model of a “two-compartment tumor metabolism” proposes that, during tumor growth, cancer cells release H2O2 and other reactive oxygen species in the tumor microenvironment [57,58]. This, in turn, is followed by a loss of Caveolin-1 protein and induction of autophagy and mitochondrial dysfunction in the stromal fibroblasts [58]. As a consequence of the reduction in the number of mitochondria, stromal fibroblasts shift their metabolism toward glycolysis and produce high-energy mitochondrial fuels, such as L-lactate, pyruvate and ketone bodies. These metabolites, released in the intercellular space, are used by neighboring, autophagy-resistant cancer cells to fuel their oxidative mitochondrial metabolism with a concomitant increase of mitochondrial mass [55]. Salem and coauthors [55] demonstrated that GOLPH3 up-regulation protects MDA-MB-231 breast cancer cells from autophagy and increases mitochondrial biogenesis. Moreover, they reported increased tumor growth when cancer cells overexpressing GOLPH3 were injected into the flasks of athymic nude mice. In agreement with these data, Nakashimi-Kamimura and coauthors showed that GOLPH3 protein shuttles between the Golgi apparatus and mitochondria, controlling their mass through a mechanism that depends on mitochondrial lipids [59]. 

## 7. Correlation of GOLPH3 Upregulation with Cancer Cell Proliferation and Tumorigenesis

**Figure 2:**
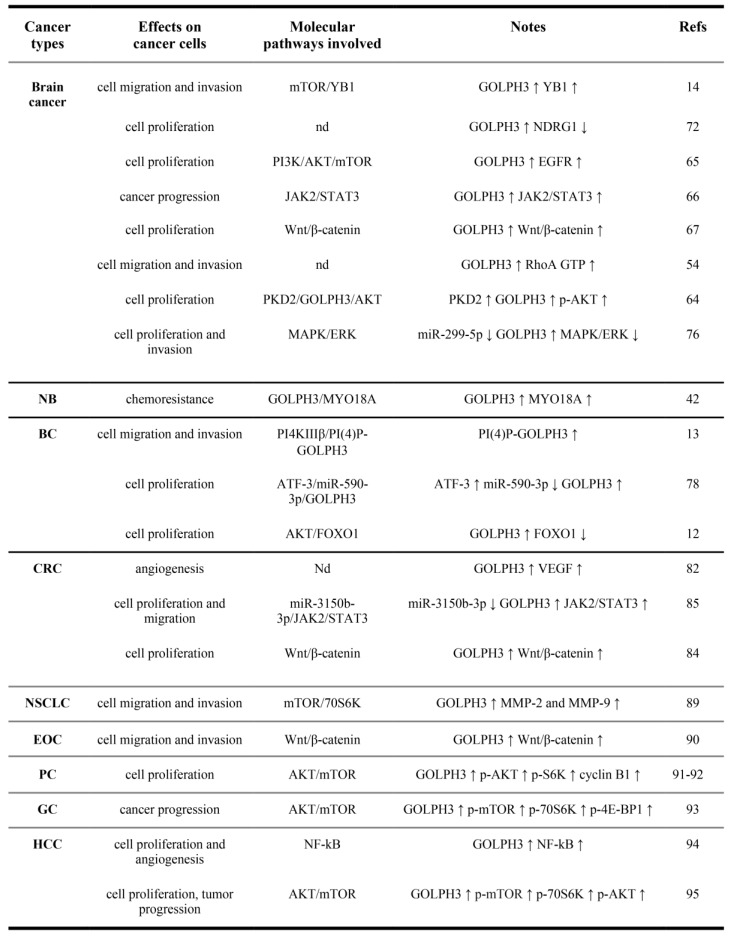
Effects of GOLPH3 up-regulation in solid tumors and molecular pathways involved. NB, Neuroblastoma; BC, Breast cancer; CRC, Colorectal cancer; NSCLC, Non-Small Cell Lung Cancer; EOC, Epithelial Ovarian cancer; PC, Prostate cancer; GC, Gastric Cancer; HCC, hepatocellular carcinoma. (↑),upregulation; (↓), downregulation. nd, not determined. (p-), phosphorylated.

Compelling evidence indicates that GOLPH3 expression correlates with cancer-associated phenotypes and with poor survival in different solid tumors. In the following paragraphs we will discuss briefly the experimental data obtained in the most-studied cancer types (Figure 2). 

### 7.1. GOLPH3 Deregulation and Brain Tumors

Primary tumors of the Central Nervous System (CNS) are a heterogeneous group of malignancies affecting mainly the brain and, to a lesser extent, spinal cord, sellar region, and cranial and spinal nerves. A recent classification by the World Health Organization (WHO) indicates that different CNS tumor entities or variants are approximately 130 [60]. Glioma is the most common tumor of the brain. The molecular pathways involved in CNS tumor formation have been partially elucidated in the last years. However, despite the growing evidence of GOLPH3 involvement in CNS tumor formation, this protein is not yet included in a standard set of their molecular biomarkers [60,61].

The role of GOLPH3 in CNS tumor etiology was first proposed by Li and collaborators in 2011 [62] who found that more than half of their patients affected by glioma are positive for the overexpression of either GOLPH3 protein or RNA and that the amount of GOLPH3 expression in the glioma patients is associated with the severity of the tumor. These data were subsequently confirmed by the work of Zhou and collaborators [15] who correlated high GOLPH3 expression and poor prognosis in glioblastoma multiforme (GBM) patients and, on the other hand, low GOLPH3 expression and significantly longer median overall survival. The downregulation of GOLPH3 has been investigated also in other reports, and it has been demonstrated that depletion of this oncoprotein in human U251 and U87 glioma cell lines leads to the reduction of glioma cell migration and invasion [54]. The same report shows that this effect might be linked to the reduction of the total level of the active form of the small GTPase RhoA, which is downstream of GOLPH3. More recent studies, performed on in vitro systems, showed that migration and invasion in GBM cells (lines U251 and U87) were also promoted through the mammalian target of rapamycin(mTOR)-Y-box binding protein-1 (YB1) pathway (Figure 2) [14]. In human glioma tissues, GOLPH3, mTOR and YB1 (one of mTOR target genes, involved in cell migration and invasion) were all upregulated and exhibited a positive correlation with each other, while a downregulation of GOLPH3 through RNAi was sufficient to lower the levels of both mTOR and YB1 in these cells, thus inhibiting their motility. Similar results were obtained by inhibiting YB1 using specific siRNA or by using INK128, an inhibitor of both mTOR complexes (mTORC1/2) [14]; in both cases, artificially increasing the GOLPH3 levels alone had no effects on cell motility, showing that the mTOR-YB1 pathway was pivotal for GBM invasiveness. Recently, Arriagada and co-workers [63] reported that knocking down GOLPH3 through RNAi can deeply alter the cell morphology of a T98G cell line of GBM. This occurs because of the actin cytoskeleton rearrangement and the reduction and dynamics of focal adhesions, which in turn affect both their motility and invasive behavior. In these cells, GOLPH3 depletion led to the reduction of the cytoplasmic tyrosine kinase FAK (focal adhesion kinase) autoactivation; thus, in the tumor line, overexpression of GOLPH3 would promote metastasis formation through FAK upregulation.

Beyond invasiveness, also cell growth in gliomas is deeply influenced by GOLPH3. Zhou and collaborators [64] reported that protein kinase D2 (PKD2) upregulation promotes glioma cell proliferation (U251 and U87 cell lines), while its downregulation causes the opposite effects. The protein levels of this kinase directly influence both GOLPH3 and AKT protein levels in a proportional way, i.e., high PKD2 levels correspond to high GOLPH3 and AKT levels, and vice versa. In this scenario, artificially inverting the proportionality of GOLPH3 with respect to the kinase caused a partial rescue of the effects of PKD2 on cell growth, indicating that (i) PKD2 is an upstream effector of GOLPH3 protein in regulating AKT activity; (ii) PKD2 could increase the level of GOLPH3 by modulating the PI3K-AKT signaling pathway in vitro; and (iii) the PKD2-GOLPH3-AKT signaling pathway might be a potential glioma therapeutic target [64]. Another piece of information about the role of GOLPH3 in glioma cell proliferation came from Zhou and collaborators [65]. They demonstrated that GOLPH3 inhibits Rab5-mediated incorporation of EGFR into late endosome, thereby activating the PI3K-AKT-mTOR signaling pathway (Figure 2). Interestingly, the same report showed that the alternative EGFR-dependent signal transduction pathway, the Ras-Raf-ERK1/2 pathway, is not affected by GOLPH3 downregulation after EGF treatment and EGFR overexpression does not affect GOLPH3 protein level; thus, there is no feedback loop between EGFR and GOLPH3 [65]. 

Further insights into the relation between GOLPH3 and EGFR in tumor progression came from the study of Wu and co-workers [66]. The complex containing the membrane receptor-associated Janus kinases (JAK) and the signal transducer and activator of transcription (STAT) signaling pathway (JAK-STAT) is a downstream effector of EGFR. In GBM, the JAK2-STAT3 complex is central in tumor progression. By analyzing the U251 and U87 cell lines, the authors showed that JAK2 and STAT3 proteins, in their active form, as well as proteins and mRNAs of both cyclin D1 and c-myc (two target genes of STAT3), all followed GOLPH3 deregulation with a positive correlation. This occurs because GOLPH3, JAK2 and STAT3 are all part of the same protein complex, possibly with GOLPH3 acting as a regulating scaffold protein. Consequently, GOLPH3 may directly affect the interaction of JAK2 and STAT3 with each other, as well as their action on target genes. Interestingly, the downregulation of STAT3 only partially blocks cell proliferation induced by GOLPH3 overexpression, possibly because of the effects of its overexpression on the PI3K-AKT-mTOR pathway [66]. Another possible pathway promoting cell growth upon GOLPH3 overexpression is the GOLPH3-Wls-Wnt axis [67]. As said above, GOLPH3 interacts with the VPS35 retromer protein [9] and Wnt/β-catenin [68,69], and consequently, Wntless (Wls, the chaperone protein of Wnt secretion) [70] are important in glioma etiology. Lu and collaborators [67] have recently demonstrated that GOLPH3 affects Wls recycling, and consequently Wnt secretion and function, promoting tumorigenesis [3]. A positive correlation of the protein levels of GOLPH3 and Wls in tumor samples was shown, and they were also positively correlated with tumor grade and negatively correlated with overall survival of glioma patients [67]. In addition, artificially forcing a negative correlation between GOLPH3 and Wls partially inhibited the tumor growth, indicating that both proteins are important for tumor development, but also that they are not the only actors involved in these phenomena. In the same work [67], the authors showed that GOLPH3 is physically associated with both Vps35 and Wls in the U251 glioma cell line and that GOLPH3 regulates Wls at the protein, but not at the transcriptional, level. These data are consistent with a model whereby GOLPH3 downregulation promotes Wls lysosome-mediated degradation. In this context, the involvement of Wnt family members (specifically, Wnt2b) in glioma progression is quite straightforward [67]. Indeed, the same report showed that the entire Wnt2b/β-catenin/Cyclin D1 signaling axis is downregulated by GOLPH3 depletion. Besides its effects on tumor growth and its action in enhancing oncogene function, GOLPH3 may also act on oncosuppressors. N-myc downstream regulated gene 1 (NDRG1) has been implicated in many cellular processes including cell cycle progression, apoptosis, differentiation, and vesicular transport; it is downregulated in primary and metastatic cancers, including glioma [71]. Loss of NDRG1 has been consistently linked to tumor progression and metastasis, as well as to poor survival of breast and prostate cancer patients. As such, NDRG1 is considered a bona fide tumor suppressor. In a recent work [72], the authors showed that GOLPH3 knockdown in U251 promotes glioma cell apoptosis and increases cellular levels of both NDRG1 and cleaved caspase 3 by ~55% and ~80%, respectively. On the other hand, NDRG1 knockdown does not affect GOLPH3 expression but lowers the level of cleaved caspase 3, suggesting that GOLPH3 increases the cleavage of caspase 3 and apoptosis of glioma cells partially via downregulating NDRG1.

All together, these data quite strongly support the view of GOLPH3 as a potential target for cancer therapy [10] particularly in glioma. Actually, in the last 2 years, some authors reported on this topic. Yuan and collaborators [73] targeted GOLPH3 through siRNA loaded on cationic liposomes, obtaining the inhibition of glioma growth in vivo, in a nude mouse model. A similar nanoparticle-mediated approach was used by Ye and co-workers, who co-delivered GOLPH3 siRNA and gefitinib (Ge, an anti-EGFR drug) in vitro (U87 and T98G glioma cell lines) and in vivo (BALB/c nude mice), obtaining growth inhibition and apoptosis induction [74]. The action of gefitinib was also investigated by Wang and collaborators, who found that both immortalized and primary glioma cells with higher GOLPH3 levels also display a higher sensitivity to gefitinib [75]. These data partly contrast with the findings of Ye and collaborators [74], described above, on the GOLPH3 levels that are coupled with the gefitinib-based therapeutic strategy. Finally, Peng and co-workers [76] demonstrated that GOLPH3 is also a direct target of miR-299-5p in GBM cell lines (T98G and A172); moreover, miR-299-5p knockdown makes GBM cells sensitive to temozolomide (TMZ) both in vitro and in vivo by impairing cell proliferation and invasion and promoting apoptosis through the inhibition of the ERK signaling pathway. This suggests that miR-299-5p regulates GOLPH3 expression (downregulation) via the MAPK/ERK pathway activation and indicates that also this small RNA may be used for depleting GOLPH3 in glioma therapy.

### 7.2. Expression of GOLPH3 is Correlated with Breast Cancer Proliferation and Metastasis

Substantial work indicates that GOLPH3 is significantly upregulated in breast cancer and breast cancer cell lines, and contributes to oncogenic phenotypes (Figure 2) [12,77]. GOLPH3 upregulation significantly correlates with the advanced clinical stage of breast cancer, poorly differentiated tumors and worse prognosis of patients [12,77]. Moreover, work from Tang and coauthors [77] demonstrated that high GOLPH3 expression is linked to resistance to chemotherapy based on anthracycline+taxane+cyclophoshamide. Zeng and coauthors mechanistically associated GOLPH3 with increased levels of cyclin D1 and FOXO1 transcriptional activity via enhanced AKT activity [12]. GOLPH3 activity was also linked to breast cancer proliferation in the activating transcription factor 3 (ATF3)-miR-590-3p pathway [78]. miRNA-590-3p regulates breast cancer cell apoptosis by controlling the expression of important regulators of tumor formation and progression, such as of JAK2, PI3K, MAPK1, and CREB [79]. ATF3 is a stress-inducible protein, associated with malignant transformation and breast cancer metastasis [80]. The paper of Song and coauthors [78] demonstrated that miRNA-590-3p can directly bind the 3′-UTR of GOLPH3 mRNA and repress GOLPH3 expression. In turn, ATF3, by repressing miR-590-3p expression, modulates the miR-590/GOLPH3 signaling pathway to promote proliferation of breast cancer cells [78]. 

Tokuda and coauthors addressed the role of Golgi-PI(4)P-GOLPH3 in cell–cell adhesion, cell-migration and metastasis of breast cancer [13]. They reported that GOLPH3 affects cell migration/invasion capacity of breast cancer cells through its ability to interact with PI(4)P. In the highly invasive MDA-MB-231 human breast adenocarcinoma cells, overexpression of wild-type GOLPH3 abolished cell–cell adhesion and enhanced invasion in a mechanism that is dependent on PI4KIIIβ, that generates PI(4)P in the Golgi. 

To investigate how the overexpression of GOLPH3 might influence its roles in the oncogenesis of breast cancer, the group of Mardones compared some of the biochemical and cellular characteristics of GOLPH3 in the two human breast cancer cell lines MCF7 and MDA-MB-231 with those of GOLPH3 in the non-tumorigenic human breast cell line MCF 10A [81]. They showed that the three breast cell lines possess biochemical distinct pools of GOLPH3 and that the GOLPH3 protein distributes differently in cytosolic and membrane-bound pools in the different breast cell lines. Moreover, the three different human breast cancer lines display differences in post-translational modifications and different avidity of GOLPH3 for PI(4)P, which might influence the tumorigenic phenotypes, including the cell–cell adhesion and enhanced cell invasion. 

### 7.3. GOLPH3 Upregulation and Chemosensitivity to Cancer Drugs in Colorectal Cancer

Recent papers linked the overexpression of GOLPH3 to colorectal cancer (CRC) (Figure 2) [82,83,84,85,86,87,88]. In particular, GOLPH3 level has been associated with the clinical stage of the disease, lymph node metastasis, infiltration degree [82,83], and poor prognosis [82,83]. Studies in human colon cancer lines indicated that upregulation of GOLPH3 promotes proliferation of colon cancer cells and inhibits apoptosis by activating the Wnt signaling pathway and enhancing the expression of β-catenin [84]. It has been further suggested that the action of GOLPH3 activates AKT, which in turn activates Wnt signaling through GSK-3β [84]. 

Zhang and coauthors associated GOLPH3 protein to miR-3150b-3p function in colorectal cancer (CRC) cells [85]. They demonstrated that miR-3150b-3p is downregulated in HCT116 and SW480 CRC cell lines and that its function inhibits CRC cell growth through the JAK2/STAT3 pathway by directly targeting GOLPH3. Recent work has provided further insight into the involvement of GOLPH3 in the resistance of colon cancer cells to chemotherapeutic drugs. Specifically, the study of Zhou and coauthors correlated GOLPH3 overexpression with resistance of HT29 colon cancer cells to cisplatin, a commonly used chemotherapeutic drug that targets DNA [86]. HT29 cells that were resistant to cisplatin displayed upregulation of GOLPH3, accompanied by upregulation of P-glycoprotein (p-gp), phosphorylated pERK1/2 and β-catenin protein [86]. Conversely, depletion of GOLPH3 downregulated the expression of P-gp, pERK1/2 and β-catenin and reversed the resistance of HT29 colon cancer cells to cisplatin. Overall, these data implicated GOLPH3 overexpression in cisplatin resistance in HT29 cells via activation of the MAPK/ERK and Wnt/β-catenin signaling pathways. The same molecular pathway was involved in GOLPH3-induced resistance of HT29 cells to 5-Fluorouracil (5-FU) [87]. However, these data are at odds with the results from Wang and coauthors [88], showing that high GOLPH3 expression was significantly associated with overall survival in patients who received 5FU-based adjuvant chemotherapy and predicts higher 5-FU sensitivity in LoVo and RKO CRC cell lines. Further studies will be required to clarify whether the divergent results reflect a different response of the specific CRC cell line to 5-FU.

## 8. Conclusions

An increasing number of papers has shown that GOLPH3 drives cancer in several other solid tumors such as Neuroblastoma (NB) [42], Non-Small Cell Lung Cancer (NSLC) [89], epithelial ovarian carcinoma [90], prostate cancer [91,92], gastric cancer [93], and hepatocellular carcinoma [94,95] (Figure 2). Recent reports indicate that GOLPH3 overexpression can be used as a positive biomarker for tumor progression and poor survival in these cancer types [89,90,91,92,93,94,95,96,97,98]. Experimental data in cancer cell lines or in nude mice revealed the role of GOLPH3 in cell proliferation, metastasis formation and angiogenesis (Figure 2). These studies have also led to elucidate the molecular pathways that involve GOLPH3 function in tumorigenesis. Substantial evidence has correlated GOLPH3 oncogenicity with enhanced AKT/mTOR signaling and Wnt/β-catenin. Recent work has linked the up-regulation of GOLPH3 protein to activated NF-kB signaling in hepatocellular carcinoma [94]. However, further studies are required to validate the involvement of this pathway in other cancer types. The relation between high GOLPH3 and poor prognosis, coupled with the recently obtained results regarding the sensitivity of cancer cells to chemotherapy in breast, colorectal and brain tumors upon its depletion (see above), strongly support the idea of GOLPH3 as a promising target for these therapeutic approaches. GOLPH3 also enhances cancer cell survival if the administered drug is a DNA damage-inducing chemical [29], thus its knockdown might be exploited in combination with these substances to enhance the efficacy of chemotherapy. Acting at the same time on several parallel pathways controlling cell proliferation, mitochondrial function and cytokinesis, and genome integrity might be a powerful approach to treat tumors overexpressing GOLPH3. Of course, further studies are required to find the optimal protocol and, eventually, to identify other possible approaches, especially from the perspective of a personalized treatment based on the molecular characteristics of each tumor considered, on a case-by-case basis. In this context, the use of model organisms such as *Caenorhabditis elegans* and *Drosophila melanogaster* will be of great help to test new molecules impairing in vivo the activity of either GOLPH3 or its molecular partners.
